# Clinical Functional Genomics

**DOI:** 10.3390/cancers13184627

**Published:** 2021-09-15

**Authors:** Seren Carpenter, R. Steven Conlan

**Affiliations:** Swansea University Medical School, Singleton Park, Swansea SA2 8PP, Wales, UK; 1907715@swansea.ac.uk

**Keywords:** functional genomics, clinical adoption, CRISPR, single cell analysis, ethnic diversity

## Abstract

**Simple Summary:**

Functional genomics refers to the activity of the genome, that is, how the information contained in DNA (the book) is read and ‘acted upon’ in a biological context. Genes are turned ‘on’ (resulting in the synthesis of RNA that is translated into proteins) or ‘off’ during development and in response to environmental stimuli. Mis-regulation of these process can manifest as disease. Functional genomics are currently being developed clinically to improve patient care, with some clear potential future goals within the field. This commentary discusses rapidly evolving clinical functional genomic pathways and the underpinning technologies that have allowed for recent research and scientific advancements, and addresses challenges faced in the field.

**Abstract:**

Functional genomics is the study of how the genome and its products, including RNA and proteins, function and interact to affect different biological processes. The field of functional genomics includes transcriptomics, proteomics, metabolomics and epigenomics, as these all relate to controlling the genome leading to expression of particular phenotypes. By studying whole genomes—clinical genomics, transcriptomes and epigenomes—functional genomics allows the exploration of the diverse relationship between genotype and phenotype, not only for humans as a species but also in individuals, allowing an understanding and evaluation of how the functional genome ‘contributes’ to different diseases. Functional variation in disease can help us better understand that disease, although it is currently limited in terms of ethnic diversity, and will ultimately give way to more personalized treatment plans.

## 1. Introduction

The human genome is arguably the most useful information we currently possess to help improve patient health and is a key to medical advancement, as whole genome sequencing (WGS) of an individual maps out their entire unique genome, making it possible to pinpoint abnormalities or monitor patient improvement as a treatment is given.

The functional genome, namely the transcriptome, proteome, metabolome and epigenome, together contribute to specifying the phenotype of an organism. Information in genes as DNA is transcribed into RNA then translated into proteins; this, plus epigenetic ‘chemical tags’ induced by environmental factors, write the biological code that causes a specific phenotype. Clinical functional genomics [1] explores how these processes influence disease development and is starting to play in increasingly important role in both diagnostic and prognostic procedures. The advent of more sophisticated technologies to identify the expression profile of RNA molecules within individual cells, and spatially resolved in diseased tissues, offers a new paradigm in both hematology and pathology. This, coupled with the advent of gene editing technologies, is leading to a new era of understanding diseases, and from which new diagnostics and therapeutics will emerge.

## 2. Main

### 2.1. Clinical Functional Genomic Pathway

Functional genomic analysis can be performed on a patient sample using high-throughput technologies like bulk RNA sequencing or newer methods, including spatial transcriptomics, to provide insight into the cellular transcriptome [2]. These are compatible with next generation sequencing (NGS) which is a massively parallel, high-throughput technology that rapidly determines the order of nucleotides in entire genomes or targeted regions [3]. It is also possible to use microarrays to profile multi-gene expression, however, while this method is more cost-effective, it does not give the same complete picture as RNA sequencing or spatial transcriptomics. If only a small number of genes need to be tested, real-time PCR provides a highly sensitive and cost-efficient method of choice. Downstream bioinformatic analysis of the patient’s unique functional genome can identify diseases or disorders, allowing for a tailored treatment plan specific to the patient. This more accurate diagnosis and treatment results in better prognosis for patients and is the fundamental basis of precision medicine.

#### 2.1.1. Current Clinical Research

The field of functional genomics is of great relevance clinically to cancer patients, where most research and development is currently focused. Changes in the genome or epigenome can cause cancer by promoting uncontrolled cell growth or causing the immune system to fail to destroy tumors. Using a clinical functional genomics approach can allow for earlier and more accurate cancer diagnosis, leading to more accurate treatment options and better prognosis for patients. The UK government is hopeful about staying at the forefront of genomic research and new discoveries using the initiative ‘National NHS Genomic Medicine Service’. In the UK, WGS has already begun to enter clinical practice and it is the government’s aim to increase this and to offer elements of the functional genomic pathway for diagnosis of cancer. Using functional genomic methods such as RNA sequencing has been shown to successfully detect relapsing cancer up to 200 days before the relapse appears on a CT scan. This success has led to RNA sequencing being introduced more routinely in cancer diagnostics [4]. Functional genomic techniques open the door to personalized medicine, especially for cancer patients, and allow doctors to form personalized treatment plans. WGS and epigenomics for an individual patient with cancer will identify genetic mutations and epigenetic alterations as circulating biomarkers; in cancers, these are circulating tumor cells (CTCs) and circulating tumor DNA (ctDNA) which can originate from the primary tumor, providing early detection. Biomarkers from metastasizing tumors are also extremely difficult to detect as they are present in such low abundance and can only be detected using highly sensitive technologies.

The Functional Genomics Centre at AstraZeneca is currently working with Cancer Research UK using clustered regularly interspaced short palindromic repeats (CRISPR) technology to study cancer biology and create better, more appropriate biological models. Alongside this, they are developing informatic processes to analyze ever-increasing dataset numbers [5]. The clinical implications are the possibility of developing more effective cancer treatments. Similarly, the company GSK joined forces with the University of California to form the Laboratory for Genomic Research (LGR). The main focus of the LGR is to use functional genomics to improve drug discovery methods using CRISPR to knock out or alter multiple genes in one experiment at scale. This allows scientists to explore the function of genetic variants quickly and simply, with the ultimate goal of new and improved drug production, leading to more novel therapies since these methods increase clinical success [6].

#### 2.1.2. Current Clinical Applications

The rapid development of functional genomics in the past two decades has allowed for breakthrough discoveries in certain cancer research, such as in acute myeloid leukemia (AML) and breast cancer studies. Novel molecular biology technologies, such as short hairpin RNA (shRNA) and CRISPR-Cas9, and advances in bioinformatics have allowed for wider research into the physiopathology of AML, resulting in a cure being within reach [7]. Furthermore, functional genomics have been used effectively to guide drug development, including one of the earliest examples; the discovery that the gene HER2 is overexpressed in certain types of breast cancers, which led to development of a drug, Herceptin [8,9]. Identifying drug targets within diseases allows for more rapid drug development or the repurposing of existing drugs. This is majorly beneficial to the pharmaceutical industry as it lowers costs and speeds up development times. If similar studies and clinical trials were to be conducted on other diseases, it could rapidly lead to deeper understanding of the diseases and improve drug discovery.

#### 2.1.3. Benefits of This Field to Science and Public Health

Variant interpretation—deciding if a genetic change is pathogenic or benign—is a common issue caused by lack of understanding of biological functions within the genome [1]. Functional genomic studies investigate biological processes and how genes, RNA and proteins interact to form phenotypes, rather than just exploring the genome per se. These processes and pathways are complicated due to the ever-changing nature of the transcriptome, epigenome, proteome and metabolome. A clinical functional genomic approach can improve this issue by linking WGS datasets with functional omics datasets. Interpretation can be improved by widening datasets, such as increasing ethnical diversity within studies. This could lead to significant clinical results for patients by improving understanding of biological processes to potentially reveal insights that were not possible to achieve with the genome alone.

#### 2.1.4. Challenges Currently Being Faced within the Field

High costs are currently one of the major factors limiting the clinical expansion of the field. Lowering the cost of sequencing whole genomes would make it more accessible and easier to integrate into healthcare systems around the world. According to pricing from the US National Human Genome Research Institute, the cost per megabase of sequence data has plateaued since 2016 and remains fairly high, at around USD 1000 per genome for sequencing alone. The cost of genome sequencing per cancer case is roughly GPB 6850 in the UK [10], therefore despite the service being made available to the NHS, certain health trusts/boards may not offer it routinely due to high costs. However, these hospitals should be encouraged to perform genome sequencing as an investment in healthcare since treatment cost are significantly lower if the cancer is diagnosed early [11]; the UK governments’ ‘Life Science Vision’ is focused on such early diagnosis and prevention [12].

Functional genomic studies should lead to equitable personalized medicine. However, a challenge faced within the research is that there is not enough population and demographic diversity. Sirugo et al. determined that most genome-wide association studies (GWAS) are performed in high income countries and the percentage of individuals in GWAS based on ethnicity were 78% European, 10% Asian, 2% African, and all other ethnicities represented 1% or less of GWAS [13]. This is not equitable representation, and this information disparity can cause clinical genome interpretation to be less reliable for the underrepresented minorities. As use of WGS and functional genomics begins to increase clinically, we must ensure that reference genomes are diverse enough to be used for all populations and ethnicities. If ethnical diversity in genome studies is not increased, this will block large groups of people from accessing this form of healthcare.

Since functional genomics looks at more than just the genome, it is currently uncertain whether some functional aspects will be tissue- or cell-dependent and therefore it is unclear how well functional data obtained from blood samples can compare directly to difficult-to-collect tissue samples, e.g., from the brain. Comprehensive functionally annotated genomes are beginning to be assembled, enabling scientists to compare vast amounts of genomic data to assess this issue in depth [1].

Most multi-omics studies have been performed on animal models rather than human cells or tissues, and this creates the issue of then translating this functional genomic data into data that are useful or applicable in human disease. This issue can be resolved in the future by performing clinical trials on humans or by using the engineered humanized physiologically relevant animal models in research studies.

Ethical restrictions impact most areas of scientific research, with this field being no exception. The rapid growth of technologies and knowledge in this field has allowed for many discoveries, however the time taken to assess risks and benefits of clinical genomic testing has and will continue to slow down research, although thoroughness is necessary to ensure the safety of patients. The discussion around the ethics of human gene editing has led to many prominent scientists insisting on banning human germline editing while research is conducted to prove it can be done safely and effectively [14]. While this does slow down progress, there is a broad societal consensus that this is an important step since the technology is not yet properly understood.

### 2.2. Functional Genomics Technology Platforms

The rapid development of new technologies over recent years has increased and continues to increase our ability to analyze the genome, transcriptome, and quantify the proteome and metabolome of single cells. Functional genomics uses single cell analysis technologies, like single cell sequencing, alongside omics datasets and high-throughput technologies, which enable simultaneous analysis of thousands of single cells, to study and understand how genetic variants can affect disease pathogenesis [12]. Other techniques such as mass spectrometry have recently reemerged as key analytical tools for proteomic and metabolomic analysis of single cells.

#### 2.2.1. Single Cell Analysis

**Figure 1:**
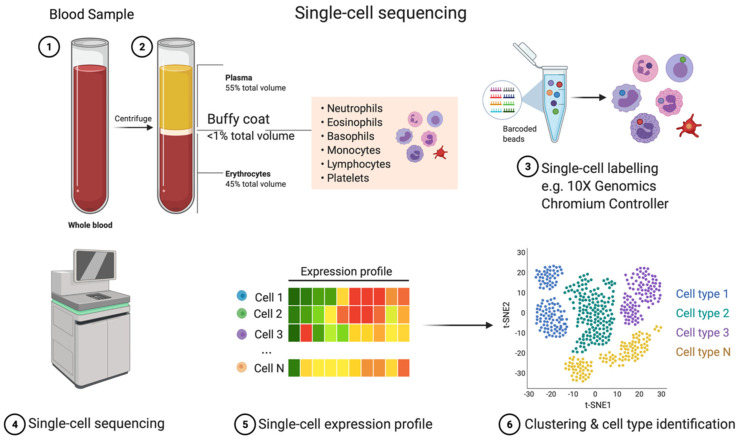
Single cell transcriptomics is a technology used to resolve RNA-seq data at a single cell level, and thereby all mRNAs (and miRNA and lncRNA where the technology permits). Cells may be derived from homogenized tissues, but also from the immune cell component (Buffy coat) from whole blood samples. This technique allows for gene activity measurements from all cells present in a sample, assuming highly efficient cell labeling. Cell samples are isolated, and each cell is individually labeled using sequencing bar code technologies, e.g., microfluidics. Labeled cells can then be re-pooled and sequenced. Finally, data are analyzed, and gene clusters are associated into cell types or tissue domains. Adapted from Single Cell Sequencing, by BioRender.com (2021). Retrieved from https://app.biorender.com/biorender-templates.

Single cell analysis (Figure 1) was developed only a decade ago in 2011 and has become majorly beneficial to the field of functional genomics. It has enabled a view of cell-specific and cell-cell interactions at a single cell level. This in-depth view of cells allows molecular profiling that previously could not be revealed and, moving forward, will be particularly beneficial in hematological cancer diagnosis and monitoring, as well as in monitoring and understanding immune responses to, for example, SARS-CoV-2 in COVID-19. A single-cell multi-omics approach is perhaps the best way forward in the characterization of cancer functional genomes as it is the only technique that can achieve full resolution of tumor heterogeneity [14].

#### 2.2.2. NanoString

NanoString is a type of DNA microarray originally developed for use in cancer diagnostics but now has many clinical applications alongside oncology, such as immunology research. The amplification-free NanoString nCounter single cell assay system is highly sensitive and measures nucleic acid content by counting molecules and directly profiling them in a highly multiplexed single reaction to profile gene expression. This eliminates amplification bias, meaning this technology presents a potentially more reliable way to analyze DNA or RNA [15]. NanoString should currently be viewed as complementary to NGS and not a full replacement in all settings [16].

#### 2.2.3. Spatial Transcriptomics

**Figure 2:**
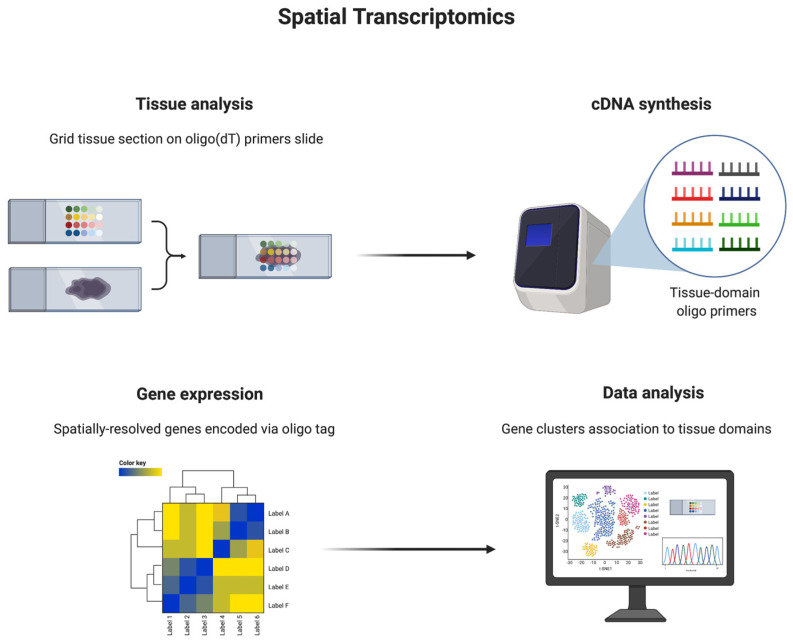
Spatial transcriptomics is a technology used to spatially resolve RNA-seq data, and thereby all mRNAs, in individual tissue sections. This technique allows for gene activity measurements and mapping in tissue samples. Tissue samples are prepared on glass slides and, by means of tissue-domain oligo primers, genes are encoded. Finally, data are analyzed, and gene clusters are associated with tissue domains. Reprinted from Spatial Transcriptomics, by BioRender.com (2021). Retrieved from https://app.biorender.com/biorender-templates, accessed on 3 September 2021.

Spatial transcriptomics (Figure 2) is a molecular profiling method that allows gene activity to be mapped across a tissue sample. The main method used to perform this is by positioning the sample on an array of spatially barcoded reverse-transcription primers that attach to mRNA with oligo(dT) tails. The library product is compatible with NGS technologies, which allows for massive transcriptional profiling. This method is incredibly useful as it gives scientists insight into an individual’s entire tissue sample which could help diagnose a disease, for example, through determining cellular heterogeneity, and even towards cancer stem cell identification [17].

#### 2.2.4. The Use of CRISPR-Cas9 within Functional Genomic Studies

**Figure 3:**
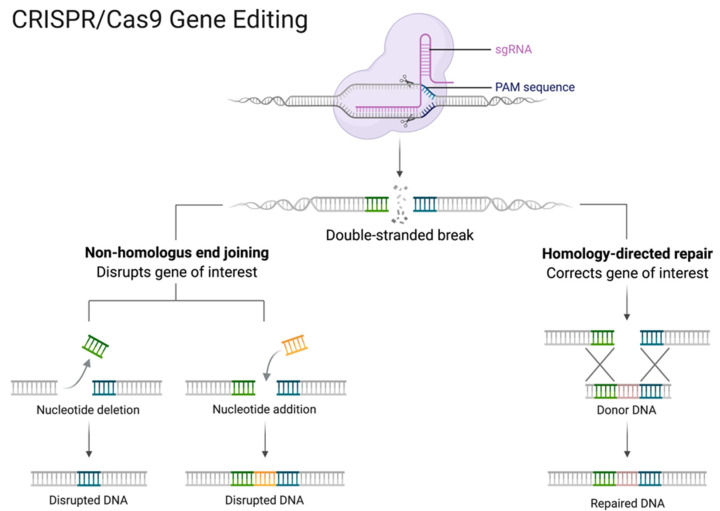
CRISPR/Cas9 is a powerful tool for genome engineering. The Cas9 complex with a sgRNA recognizes a specific sequence, the protospacer. This is only possible if this sequence is followed by a Protospacer Adjacent Motif (PAM). When Cas9 binds, a dsDNA break is generated. Then, non-homologous end joining or homology-directed repair can occur, leading to mutations or gene changes, respectively. Reprinted from CRISPR/Cas9 Gene Editing, by BioRender.com (2021). Retrieved from https://app.biorender.com/biorender-templates, accessed on 3 September 2021.

CRISPR-Cas9 (Figure 3) is a revolutionary discovery that allows for the editing of genes that have been found to be, for example, mutated in diseases, though studying such mutations in model systems using a functional genomics approach will allow the consequences of genetic mutations to be determined. From this, approaches that will change the way we treat certain diseases and identify drug targets will be discovered.

CRISPR edits genes by releasing guide RNA (gRNA) that targets and attaches to a specific section of DNA within the genome. This allows the enzyme Cas9 nuclease to cut the DNA at that section which activates the cell’s own DNA repair process. Sequences within the cut gene can be edited or deleted and replaced with a new DNA sequence. This process is quick and easy, making it very cost-efficient. The ability to edit genes quickly and inexpensively allows for experiments to determine causes of diseases that were previously unknown [5]. CRISPR-Cas9 could become a key part of the genetic screening industry as it can be used to engineer embryos, although there are many ethical debates surrounding this topic currently. All programmable nucleases, including CRISPR nucleases, are in the process of being clinically investigated to gather enough evidence to suggest whether they are safe and effective. If they are proven to be effective and safe in clinical trials, they could be used to treat patients with a wide variety of diseases, from hereditary blindness to cancers.

Despite the potential benefits, there are issues with the programmable nucleases that have come to light during clinical trials. They can cause unwanted and dangerous mutations; this is a big issue with regards to using the technology with humans, as mutations may contribute to oncogenesis. The enzyme Cas9 can be immunogenic, therefore initial clinical trials have only been conducted in an immunologically privileged organ, the eye. Certain editing approaches have started to be developed that appear to overcome some of the challenges of working with nucleases to edit genomes. These editors use a Cas9 nickase, which unlike a wild-type nuclease produces DNA single-strand breaks, not double-strand breaks. This means the nickase editors are unlikely to cause large deletions or chromosomal rearrangements during the DNA repair process. Ultimately, this engineered gene editing nuclease appears to work more reliably and efficiently at correcting genes. Due to the reduced risk of unwanted mutations, these base and prime editors would be ideal for germline or in utero editing in the future if these processes become legal. These processes could eventually be used to correct pathogenic mutations in human embryos, since gene editing in newborns is generally inefficient. This ethical discussion may come around sooner than expected as society is always looking for ways to improve public health, both after and potentially even before birth. However, it is important not to rush this process as, despite the promising results of current base and prime editors, they can still be improved [14].

When CRISPR-Cas9 are developed enough to resolve all issues and the process is safe for use in humans, this could become a pivotal part of the clinical functional genomic pathway and be especially useful to the genetic screening industry because in the future in utero editing may be favored over preimplantation genetic diagnosis since it will not require destruction of human embryos, although this will remain a highly controversial approach.

### 2.3. The Functional Genomic Market

The success and importance of genome sequencing during the COVID-19 pandemic drew government attention to the already rapidly expanding field. This, along with increasing cancer cases, resulted in more government interest and funding, such as in the UK with the launch of the GBP 200 m life science investment program in summer 2021. This investment is expected to generate around GBP 600 m long-term capital for the industry in the UK [12].

North America accounted for the largest share of the genomics market in 2020, with established companies such as Illumina, Inc. and Thermo Fisher Scientific dominating the market [18]. The main driving force of this market is the sequencing technology: This segment accounted for the largest technology share of the genomics market in 2019 due to its rapid advancements and usefulness to a wide variety of sectors. The drug discovery and development sector, which uses many of these technologies, accounted for the largest share by application of the genomics market in 2019 [19].

The value of the global genomics market is expected to more than double by 2025 [19] and could create around 133,000 jobs by 2030 [20]. This may cause issues within the industry, as there is already a shortage of trained professionals [19], unless companies start investing now in training more scientists in this area to fulfill the demand.

### 2.4. Future Goals

Drug production and diagnostics are currently still developed in ‘traditional’, ways with a relatively small number of businesses such as GSK, Novartis and AstraZeneca beginning to experiment with and integrate functional genomic methods into their workflows. However, functional genomics is likely to become an essential part of all drug discovery and development pathways in the near future. The main hinderance during drug production for pharmaceutical companies is that over 50% of drugs fail at phase III clinical trials due to lack of efficacy [21]. Failures are costly and time consuming for the industry. Functional genomic techniques appear to improve success rates during drug discovery trails by mapping out potential targets within a disease, and targeting these generally results in more efficient drugs, therefore higher clinical trial success rates ultimately will lower the time taken to discover and produce vital drugs.

## 3. Conclusions

To date, functional genomics is not being used to its full potential for personalized medicine. More clinical investigations should be performed using functional genomic pathways to increase our understanding of genes and diseases and allow this knowledge to become integrated into mainstream medicine, as it is very much needed. Personalized medicine using functional genomics and genomics to determine the most effective medication at the right time based on an individual’s unique profile will transform medicine since molecular changes precede clinical manifestations; therefore, we can treat patients earlier, resulting in better and more accurate prognosis than ever before. 

Clinical trials should be performed using functional genomics to track patient progress when different drugs and treatments are used. This will deepen the understanding of diseases and how treatments affect them by measuring molecular changes compared to clinical phenotypes.
